# Measuring care coordination in German primary care – adaptation and psychometric properties of the Medical Home Care Coordination Survey

**DOI:** 10.1186/s12913-021-07100-0

**Published:** 2021-10-21

**Authors:** Aleida Ringwald, Katja Goetz, Jost Steinhaeuser, Nina Fleischmann, Alexandra Schüssler, Kristina Flaegel

**Affiliations:** 1 Institute of Family Medicine, University Hospital Schleswig-Holstein, Campus Lübeck, Ratzeburger Allee 160, 23538 Lübeck, Germany; 2 Division Nursing and Health, Department V - Social Welfare and Health, Hannover University of Applied Sciences and Arts, Blumhardtstraße 2, 30625 Hannover, Germany; 3 Federal Association for Health and Academy for Social Medicine Lower Saxony, Fenskeweg 2, 30165 Hannover, Germany

**Keywords:** Primary health care, Quality of health care, Health care quality assurance, Care coordination, Organization and administration

## Abstract

**Background:**

Continuity of care is associated with many benefits for patients and health care systems. Therefore measuring care coordination - the deliberate organization of patient care activities between two or more participants - is especially needed to identify entries for improvement. The aim of this study was the translation and cultural adaptation of the Medical Home Care Coordination Survey (MHCCS) into German, and the examination of the psychometric properties of the resulting German versions of the MHCCS-P (patient version) and MHCCS-H (healthcare team version).

**Methods:**

We conducted a paper-based, cross-sectional survey in primary care practices in three German federal states (Schleswig-Holstein, Hamburg, Baden-Württemberg) with patients and health care team members from May 2018 to April 2019. Descriptive item analysis, factor analysis, internal consistency and convergent, discriminant and predictive validity of the German instrument versions were calculated by using SPSS 25.0 (Inc., IBM).

**Results:**

Response rates were 43% (*n* = 350) for patients and 34% (*n* = 141) for healthcare team members. In total, 300 patient questionnaires and 140 team member questionnaires could be included into further analysis. Exploratory factor analyses resulted in three domains in the MHCCS-D-P and seven domains in the MHCCS-D-H: “link to community resources”, “communication”, “care transitions”, and additionally “self-management”, “accountability”, “information technology for quality assurance”, and “information technology supporting patient care” for the MHCCS-D-H. The domains showed acceptable and good internal consistency (α = 0.838 to α = 0.936 for the MHCCS-D-P and α = 0.680 to α = 0.819 for the MHCCS-D-H).

As 77% of patients (*n* = 232) and 63% of health care team members denied to have or make written care plans, items regarding the “plan of care” of the original MHCCS have been removed from the MHCCS-D.

**Conclusions:**

The German versions of the Medical Home Care Coordination Survey for patients and healthcare team members are reliable instruments in measuring the care coordination in German primary care practices. Practicability is high since the total number of items is low (9 for patients and 27 for team members).

## Background

Continuity of care is associated with benefits for patients and health care systems such as increased patient satisfaction, greater adherence to medical advice, decreased use of hospital services and even lower mortality rates [1]. Care coordination as one important aspect of continuity of care is defined as “the deliberate organization of patient care activities between two or more participants (including the patient) involved in a patient’s care to facilitate the appropriate delivery of health care services. Organizing care involves the marshalling of personnel and other resources needed to carry out all required patient care activities, and is often managed by the exchange of information among participants responsible for different aspects of care” [2].

The Patient-Centered Medical Home (PCMH) as an example for a model of delivering primary care aspires to expand the existing primary health care in the following five functions: comprehensiveness of care, patient-centered health care, coordination of care, accessibility of services and quality and safety [3, 4]. The PCMH has been implemented in the US-American health care system and showed promising results related to clinical outcomes, utilization of health care services and costs [5, 6]. In 2009, based on international and national primary care concepts the German Advisory Council on the Assessment of Developments in the Health Care System proposed the model of the “Primary Care Practice” as a future-oriented, population-based concept in German primary care [7]. Key feature is a team-based, patient-centered approach containing elements of the PCMH in order to resolve shortcomings of the German health care system that were all attributed to various coordination challenges [7]. In 2019, care coordination deficits were still visible in German primary care in comparison to ten other countries [8]. In particular the exchange of relevant information about patients between primary care physicians and specialists and the information technology that facilitates care coordination with patients and other clinical providers have potential for improvement [8].

In Germany, primary care of the adult population is delivered by specialists in family medicine, specialists in internal medicine and general practitioners without further specialization [9]. The German health system allows patients to choose their physicians in primary as well as in secondary care [10]. A contact with the primary care physician to get access to secondary care is not necessary, which complicates care coordination efforts. However, there are approaches to foster the gate keeping role of primary care physicians. The “general practitioner centered care” stipulates that referrals to medical specialists in secondary care “are preceded by relevant diagnostic procedures and treatments [at the primary care physician] and, in case of referral, the findings are clearly communicated to medical specialists and backwards” [11]. Participation in this program is voluntary for physicians and patients, and comes with an about 40% additional reimbursement for enrolled patients [11].

Physicians working in German primary care are usually self-employed in individual or joint practices or medical treatment centers [12], whereat office-based single handed primary care physicians dominate [10]. It is reported that primary care physicians in Germany experience noticeable time constraints during patient visits, which further impedes the task of care coordination [13]. Thus, German primary care physicians were found to ask less questions and give less advice than physicians in Great Britain or the US [14, 15]. Consistently, primary prevention approaches (e.g. lifestyle counseling) are probably insufficient established in primary care [16]. German primary care physicians are supported by non-physician health professionals in their practice, which are comparable to medical assistants in the US [17]. Facing more complex health needs of patients, their fields of responsibility have been extended from administrative and simple medical tasks to more complex tasks including disease and care management over the last years, provided that additional training is completed [17].

Positive experiences regarding care coordination and access to care received by primary care physicians led to a higher satisfaction with care in German adults [18]. Moreover, a qualitative study showed that patients perceive coordination and continuity of care as very important [19]. Care coordination showed to have positive effects on health-related functional status and the reduction of hospital readmission and healthcare costs [20, 21]. This applies in particular to patients with chronic conditions who benefit from a strong primary care system [22, 23]. However, coordinating the appropriate care for the patient’s needs in the health care system requires good knowledge of the patient and the system itself [24].

For improvement, care coordination needs measurement. Suitable tools must cover for instance different aspects of communication, transfer of information and transition between different health sectors to meet the multidimensional concept. The “Care coordination Measures Atlas” published by the Agency for Healthcare Research and Quality (AHRQ) encompasses a range of tools for evaluating several domains of care coordination [25]. A systematic review of the care coordination landscape showed that none of the included instruments was able to cover all aspects of care coordination defined by the AHRQ [2, 26].

Due to the deficits of the available tools the Medical Home Care Coordination Survey (MHCCS) was developed, containing a patient (MHCCS-P) and a healthcare team version (MHCCS-H) to measure care coordination from both perspectives [27]. The authors of a review that explored tools which evaluate progress during PCMH-transformation concluded that the MHCCS-H was the only instrument out of five that also included items on the comprehensiveness of care, e.g. MHCCS-H item “the primary care practice has behavior change interventions readily available for patients as part of routine care” [28]. Comprehensiveness of care describes “the breadth of services a practice offers to address any health problem at any given stage of patient’s life” [28].

The aim of this study was the translation and cultural adaptation of the MHCCS into German and the examination of the psychometric properties of the resulting German versions of the MHCCS-P and MHCCS-H in order to provide an instrument that is able to measure and, consequently, optimize care coordination in German primary care.

## Methods

### Study design

The study was carried out as a cross-sectional, paper-based survey.

### Translation and cultural adaptation

The translation and cultural adaptation followed the Principles of Good Practice for the Translation and Cultural Adaptation Process by the International Society for Pharmacoeconomics and Outcomes Research (ISPOR) task force [29]. After obtaining permission from the first author of the original publication for the development and validation of the MHCCS, Ianita Zlateva from the Weitzman Institute, USA, two postgraduate family medicine trainees independently translated the English versions of the MHCCS-P and MHCCS-H into German. The results were discussed during two consensus telephone meetings with other researchers in an interdisciplinary team of two physicians (JS, KF), a medical student (AR), a nursing scientist (NF) and a health scientist (AS) in order to reconcile the forward translations into a single forward translation of both the MHCCS-P and the MHCCS-H. The consented versions were back translated. In a back translation review between back translator and one researcher (KF) discrepancies were discussed and solved. The back translations with explanatory notes were sent to Ianita Zlateva who approved those versions. The cognitive debriefing was successfully executed in one primary care teaching practice of the Institute of Family Medicine in Lübeck, Germany. In this teaching practice, the instruments were tested on a small group of respondents from the target populations – all health care team members and patients that were present at the time of the researcher’s practice visit were invited to participate - to check for understandability, cultural relevance and interpretation of the translation [29]. A researcher team (JS, KF, AR) followed through the review of the cognitive debriefing results in order to finalize the German version.

### Recruitment and data collection

The recruitment was realized as a convenience sample of teaching and research practices of the Lübeck Institute of Family Medicine in Schleswig-Holstein as well as primary care practices approached by the doctoral candidate AR in Hamburg and Baden-Württemberg. Four primary care practices took part in the healthcare team member survey, whereas eight primary care practices took part in both the healthcare team member survey and the patient survey. Additionally, members of a network of primary care practices in Northern Germany and primary care practice assistants of a continuing education course were invited to participate in the healthcare team member survey. The aim were sample sizes of about 232 patients and 164 healthcare team members as these were the numbers used in the original validation study [27].

Within participating primary care practices, inclusion criteria for both patients and healthcare team members were a minimum age of 18 years and a sufficient knowledge in the German language. Within the interval of one week all patients were invited by the practice team to participate in the survey. The healthcare team included everyone who was involved in the patient’s care to facilitate the appropriate delivery of health care services [2].

Each participant received a paper-based questionnaire and an unlabeled envelope to deposit the completed questionnaire. The envelopes were put into a box, which was sent to the Institute of Family Medicine by the practice team for data analysis. Data collection took place from May 2018 until the end of April 2019.

### Measures

The MHCCS-P and MHCCS-H are validated, multidimensional instruments, which assess the structures behind care coordination in primary care practices from the perspective of the patient as well as from the perspective of the healthcare team member [27].

### MHCCS-P

The original patient version (MHCCS-P) contains 13 items out of four care coordination domains (plan of care, communication, link to community resources and care transitions).

To quantify the convergent construct validity of the German MHCCS-P the established German version of the European Project on Patient Evaluation of General Practice Care (EUROPEP) was added to the MHCCS-P [30, 31].

The EUROPEP is an internationally validated, multidimensional and established instrument with 23 items measured on a 5-point Likert scale from “poor” to “excellent”. The items are represented by the two dimensions “clinical behaviour” (items 1–16) and “organisation of care” (items 17–23) [32].

The final German version of the patient questionnaire used for this survey consisted of 48 items: 13 items representing the German MHCCS-P measured on 5-point Likert scales (“always”/“agree” to “never”/“disagree”), two overall rating items of received health care and felt care coordination (“very good”/“agree” to “very bad”/“disagree”), four yes/no-questions in between, six sociodemographic items, and 23 German EUROPEP items. Participants could add their comments in a free text box.

### MHCCS-H

The team version (MHCCS-H) of the original MHCCS includes 32 items covering eight care coordination domains (Accountability, IT capacity, Follow-up Plan of Care and Self-management in addition to those of the MHCCS-P) [27]. The final German version of the healthcare team member survey included 44 items: 35 items representing the German MHCCS-H, one yes/no-question in between, one overall rating of practice’s care coordination (“very good” to “very bad”) and seven additional items asking for sociodemographic data. Healthcare team members could add their comments in a free text box as well.

### Statistical analysis

Statistical analysis was performed using SPSS 25.0 (Inc., IBM). The descriptive analysis of sociodemographic data delineated frequencies with means and standard deviations for the patient and the team survey.

The assessment of psychometric properties comprised an item analysis including descriptive statistics of mean, standard deviation, variance, skewness and kurtosis as well as item difficulty. Values from 20 to 80% are considered preferable [33]. The corrected item-total correlation was calculated in order to assess whether the individual items measure the same construct as the other items as a whole. Correlation values less than 0.3 specified items that did not correspond well with the overall scale [34].

Exploratory factor analyses were carried out to test the construct validities of the MHCCS-P and MHCCS-H. Due to a high number of missing values the approaches with listwise deletion of cases with missing values and the replacement of missing values with the variable mean were compared for the MHCCS-P. In order to test the dimensionality of the domains a principal component analysis (eigenvalue> 1 or retention of a specified number of factors in consideration of the original domain structure, varimax rotation) with extraction of component loadings was performed. Sample suitability was evaluated with the Kaiser-Meyer-Olkin (KMO) criterion. Additionally, the measure of sampling adequacy (MSA) for each item was included in the evaluation of the analysis. Bartlett’s test was used for examining sphericity [34]. The variances of the variables that has been accounted for by the extracted factors (R^2^) were considered. By showing component loadings λ > 0.3, the items were assigned to that particular component. Reliability was described with Cronbach’s α as a measure for the internal consistency [35].

Convergent, discriminant and predictive validity were calculated using Spearman’s rank order correlation. To determine if the expected measures correlated with the domains convergent validity was tested by correlating the general care given by the primary care practice team with all domains of the German MHCCS-H. For calculating the convergent validity for the German MHCCS-P the resulting domains from factor analysis were correlated with the two EUROPEP dimensions “clinical behaviour” and “organisation of care” [32]. In line with the authors of the original instrument development discriminant validity was calculated by correlating the gender of the team members with the domains for the MHCCS-H and the gender of the patients with the domains of the MHCCS-P to show that no relationships existed with non-related concepts. Thus, the predictive validity, which examines if the instrument is able to predict other important outcomes, was calculated for the MHCCS-P by correlating the domains of the MHCCS-P with both the self-reported general health status and the self-reported overall coordination of care. Correlations between 0.4 and 0.6 represented good correlations [36].

An alpha level *p* ≤ 0.05 was used for tests of statistical significance.

### Ethical approval

Ethical approval was given by the ethics committee of the University of Lübeck (No 17–374) on January 10, 2018. The return of the completed anonymous, paper-based questionnaire in a closed envelope was classified as informed consent.

## Results

### Translation and cultural adaptation

During the translation process, the expression “the primary care team” was changed into “our primary care team” in the MHCCS-H in order to clarify which team is meant, to make the team members feel more involved and to refer to the style of the patient questionnaire using “my primary care team” throughout the questionnaire. Item 3 of the MHCCS was changed from “The primary care team is characterized by collaboration and trust” to “… characterized by trusting collaboration” to ask specifically for one concept instead of two in one item. Item 5b “The primary care team uses electronic data to monitor and track patient health indicators and outcomes” was divided into two items “…monitor the outcomes of patient treatment” and “…track quality indicators of patient care…”. The item 7c “The primary care team considers and respects patients’ values, beliefs and traditions when recommending treatments” was changed into “Our primary care team considers and respects patients’ individual values when recommending treatments”, since the German word for “individual values” was considered covering “beliefs and traditions” as well.

As German survey response scales usually begin with the positive answer and end with the negative one matching the grading system in school education, all scales were used in reverse order in the German version. Furthermore, since “financial services” as support for patients were not considered as relevant as in the US we deleted this phrase from the example list of support options in item 6a of the patient questionnaire and item 15 of the MHCCS-H. Finally, clearly defined roles in health care teams such as patient self-management education, proactive follow-up and resource coordination were not explicitly known in Germany. Consequently, the consensus team decided to refrain from giving examples of clearly defined roles in item 1 of the MHCCS-H.

Eight patients (four female, four male) completed the consensus version of the German MHCCS-P while cognitive debriefing. It showed that further definitions, e.g. for “chronic disease”, were needed for the patient questionnaire. The response option “This doesn’t apply to me” was added for questions concerning the support needed while having a chronic disease, e.g. “receiving information about rehabilitation programs”. The German MHCCS-H was tested within the cognitive debriefing process by three physicians, seven practice assistants and one practice assistant trainee, overall eleven out of 15 health care team members of this primary care practice. Terms as “care plan” and “electronic data” needed more explanation, which was then added to the questionnaire.

### Sociodemographic data of participants

Out of 800 provided patient questionnaires, 350 were returned by participating primary care practices (response rate: 43%). For further analysis, 300 questionnaires were included; seven participants did not meet inclusion criteria and 43 did not visit the primary care practice in the last twelve months (first yes/no-question in the instrument). More than half of the respondents were female (*n* = 163, 54%). The average age was 55 years, and 149 (50%) declared that they had a chronic disease.

In total, 410 team surveys were provided for participating primary care practices and the network of primary care practices, whereof 141 were returned (response rate: 34%). Out of 140 included questionnaires, the majority was female (*n* = 119, 85%); the mean age was 41 years (SD 13.2). Job title was provided by 135 participants in a multiple-answer-question resulting in 156 job titles, whereof 89 were practice assistants (57%), 31 primary care physicians (20%) and 36 other job titles (23%), e.g. practice manager or practice assistant with additional qualification. Further details are shown in Table 1.

**Table 1:** Sociodemographic characteristics of participating patients (*n* = 300) and healthcare team members (*n* = 140)

	Patients n (%)	Healthcare team members n (%)
**Gender**		
Female	163 (54.3)	119 (85.0)
Male	126 (42.0)	15 (10.7)
Missing	11 (3.7)	6 (4.3)
**Chronic Disease**		
Yes	149 (49.7)	
No	74 (24.7)	
I don’t know	12 (4.0)	
Missing	65 (21.7)	
**Job Title**		
Practice manager		7 (4.5)
Physician		31 (19.9)
Practice assistant		89 (57.1)
Additionally qualified practice assistant		13 (8.3)
Case manager		1 (0.6)
Administrative assistant / Secretary		5 (3.2)
Medical data assistant		1 (0.6)
Nurse		2 (1.3)
Other		7 (4.5)
**Duration of employment in participating practice**		
≤ 5 years		54 (38.6)
> 5–10 years		29 (20.7)
> 10–15 years		16 (11.4)
> 15–20 years		12 (8.6)
> 20 years		16 (11.4)
Missing		13 (9.3)
**Care Plan**	**Do you have a written care plan?**	**Does your practice make written care plans for your patients?**
Yes	63 (21.0)	43 (30.7)
No	232 (77.3)	88 (62.9)
Missing	5 (1.7)	9 (6.4)
**In the last 12 months, have you done any lab tests, like blood tests and x-rays?**		
Yes	255 (85.0)	
No	36 (12.0)	
Missing	9 (3.0)	
**How long ago were you hospitalised, if at all?**		
Less than 1 month ago	17 (5.7)	
Between 1 and 6 months ago	32 (10.7)	
Between 6 and 12 months ago	32 (10,7)	
I was not hospitalised in the last 12 months	207 (69.0)	
Missing	12 (4.0)	
	**Mean (SD**^**1**^**)** **Minimum/Maximum**	**Mean (SD**^**1**^**)** **Minimum/Maximum**
**Age**	55.1 (19.13) 18/91	41.3 (13.19) 18/66
**Duration of employment in participating practice**		9.9 (9.35) 0.1/43.0

^1^ Standard deviation

### Determination of the psychometric properties of the German MHCCS-P

The descriptive analyses of the items showed means between 1.23 and 2.08 and item difficulties ranging from 5.75 to 27%. The item “my primary care team follows through with the care plan it creates with me” showed lowest variance. “My primary care team helps me to plan ahead so I can take care of my health even when things change or unexpected things happen” exhibited conspicuous skewness (3.349) and kurtosis (13.914). Corrected item-total correlation ranged from 0.341 to 0.933. The lowest corrected item-total correlations were seen for the items of the original domain “plan of care” (0.341 to 0.580). Further details are reported in Table 2.

**Table 2:** Descriptive statistics of the German MHCCS-P

Item	Original domain	N	Mean	SD	Variance	Skewness	Kurtosis	Item difficulty in %	Corrected item-total correlation*
My primary care team asks for my ideas when we make a plan for my care.	Plan of Care	54	1.76	0.989	0.979	1.360	1.444	19.00	0.580
My primary care team follows through with the care plan it creates with me.		57	1.32	0.540	0.291	1.500	1.414	8.00	0.549
My primary care team helps me to plan ahead so I can take care of my health even when things change or unexpected things happen.		59	1.29	0.696	0.485	3.349	13.914	7.25	0.341
Someone from the primary care team helps me set goals for taking care of my health.		51	1.71	0.944	0.892	1.524	2.312	17.75	0.424
Someone in my primary care team asks me about what I need for support, e.g. with home care, equipment or transportation.	Link to Community Resources	97	1.94	1.337	1.788	1.156	< 0.001	23.50	0.860
Someone in my primary care team gives me information about services offered at their practice or in my community, e.g. counselling centres, support groups and rehabilitation programmes.		140	1.83	1.217	1.481	1.355	0.697	20.75	0.933
Someone in my primary care team encourages me to attend programs in my community that could help me, e.g. support or sports groups.		154	2.08	1.331	1.772	0.934	−0.430	27.00	0.813
I get the results of my lab tests in a timely manner.	Communication	254	1.34	0.746	0.557	2.867	9.348	8.50	0.691
Someone in my primary care team tells me all my test results, good and bad.		249	1.33	0.796	0.634	2.851	8.299	8.25	0.796
Someone in my primary care team helps me understand my test results, e.g. laboratory values or X-ray results.		251	1.40	0.805	0.649	2.395	5.871	10.00	0.625
After I leave the hospital, my primary care team knows about the care I received from the hospital.	Care Transitions	79	1.29	0.754	0.568	3.145	10.473	7.25	0.756
After I leave the hospital, my primary care team helps me to get back on my feet.		78	1.40	0.858	0.736	2.290	4.881	10.00	0.777
After I leave the hospital, my primary care team knows about new prescriptions or if there was a change in my medication.		78	1.23	0.643	0.414	3.057	9.134	5.75	0.626

*observed within original domain

It was not possible to conduct a factor analysis for the German MHCCS-P including all items and listwise deletion of cases. A factor analysis of all items with the replacement of missing values with the variable mean showed KMO = 0.721 and Bartlett’s test = 0.261. MSA were lowest for the items of the original domain “plan of care” (0.619 to 0.653). Hence, we decided to delete those items from factor analysis supported by the findings from descriptive analysis (low variance, extreme skewness and kurtosis, low item-total correlations). The factor analysis with listwise deletion of cases with missing values resulted in 29 observations included in analysis. Extracting eigenvalues over one resulted in a two-factor-solution with the items of the original domain “care transitions” loading on both factors equally. Because of this observation, the theoretical background of the original domain structure and the consideration of the scree plot we forced the extraction of three factors. The original domains (“link to community resources”, “communication” and “care transitions”) could be confirmed. The item “after I leave the hospital, my primary care team knows about new prescriptions or if there was a change in my medication” loaded on all three factors (λ = 0.462, λ = 0.487 and λ = 0.479) and was assigned to its original domain. In the factor analysis with the replacement of missing values with the variable mean each of the nine items loaded high on only one of the three factors (λ = 0.732 to λ = 0.908). MSA and R^2^ were high for both approaches and all items. Cronbach’s α ranged between 0.838 and 0.936 for the three domains. Details are shown in Table 3.

**Table 3:** Structure of the final 3 domain German patient survey as emerged from analysis (9 items, Cronbach’s α = 0.917)

Domain	Items	Factor analysis with listwise deletion of cases with missing values, ***N*** = 29, retention of 3 factors, KMO = 0.787, Bartlett’s test < 0.001			Factor analysis with replacement of missing values with the variable mean, ***N*** = 279, eigenvalue > 1, KMO = 0.739, Bartlett’s test < 0.001		
		MSA	λ	R^**2**^	MSA	λ	R^**2**^
**Link to Community Resources (α = 0.936)**	Someone in my primary care team asks me about what I need for support, e.g. with home care, equipment or transportation.	0.826	0.869	0.884	0.713	0.883	0.800
	Someone in my primary care team gives me information about services offered at their practice or in my community, e.g. counselling centres, support groups and rehabilitation programmes.	0.807	0.874	0.935	0.683	0.908	0.835
	Someone in my primary care team encourages me to attend programs in my community that could help me, e.g. support or sports groups.	0.850	0.845	0.826	0.874	0.775	0.665
**Communication (α = 0.838)**	I get the results of my lab tests in a timely manner.	0.758	0.859	0.925	0.714	0.853	0.767
	Someone in my primary care team tells me all my test results, good and bad.	0.739	0.877	0.854	0.676	0.903	0.845
	Someone in my primary care team helps me understand my test results, e.g. laboratory values or X-ray results.	0.807	0.816	0.913	0.818	0.766	0.648
**Care Transitions (α = 0.843)**	After I leave the hospital, my primary care team knows about the care I received from the hospital.	0.747	0.898	0.907	0.702	0.896	0.811
	After I leave the hospital, my primary care team helps me to get back on my feet.	0.732	0.869	0.924	0.710	0.894	0.822
	After I leave the hospital, my primary care team knows about new prescriptions or if there was a change in my medication.	0.824	0.479	0.680	0.826	0.732	0.666

The convergent validity was acceptable with r_rho_ varying between 0.329 and 0.485. It proved that the “clinical behaviour” and the “organisation of care” of the EUROPEP correlated significantly with all domains of the MHCCS-P. Further details are displayed in Table 4.

**Table 4:** Correlation of “clinical behaviour” and “organisation of care” of the EUROPEP with the domains of the MHCCS-P.

		Communication	Link to Community Resources	Care Transitions
Clinical Behaviour	Correlation coefficient	0.479	0.485	0.358
	*p*-value	<0.001	<0.001	0,001
	N	252	171	79
Organisation of care	Correlation coefficient	0.380	0.409	0.329
	*p*-value	<0.001	<0.001	0.004
	N	240	164	75

The discriminant validity was not significant with r_rho_ between − 0.139 and − 0.029 (p between 0.231 and 0.713), i.e. the gender of the patients was not correlated with the domains.

The predictive validity was acceptable and significant (*p* < 0.05) for the self-reported general health correlating with the domains “link to community resources” (r_rho_ = 0.163, *p* = 0.032) and “communication” (r_rho_ = 0.124, *p* = 0.049) but not significant for the domain “care transitions” (r_rho_ = 0.109, *p* = 0.335). The predictive validity for the self-reported coordination of care was acceptable and correlated with all domains with r_rho_ between 0.523 and 0.683 (*p* < 0.001).

### Determination of the psychometric properties of the German MHCCS-H

The descriptive analysis of the items showed means between 1.24 and 3.71. Item difficulties’ values ranged from 6.00 to 67.75%. Corrected item-total correlation showed values from 0.288 to 0.811. Missings were high (> 30%) for the items of the original domains “plan of care” and “follow-up plan of care” since 88 of 140 participants denied that their practice made care plans for their patients. More information are displayed in Table 5.

**Table 5:** Descriptive statistics of the German MHCCS-H

Item	Original domain	N	Mean	SD	Variance	Skewness	Kurtosis	Item difficulty in %	Corrected item-total correlation*
Our primary care team is made up of members with clearly defined roles in caring for patients.	Accountability	139	1.52	0.871	0.759	1.980	3.911	13.00	0.446
Our primary care team and the patients share responsibilities in managing patients’ health.		136	1.47	0.740	0.547	1.885	4.380	11.75	0.455
Our primary care team is characterised by trusting collaboration.		139	1.32	0.616	0.380	2.491	8.978	8.00	0.453
Our primary care team works with patients to help them understand their roles and responsibilities in their care.		139	1.37	0.617	0.381	1.807	3.758	9.25	0.630
Our primary care team uses electronic data (e.g. diagnostical findings, lab results), to identify patients with complex health needs (e.g. multimorbity).^N^	IT capacity	130	1.53	0.728	0.530	1.605	3.636	13.25	0.515
Our primary care team uses electronic data (e.g. diagnostical findings, lab results), to monitor the outcomes of patient treatment.		137	1.36	0.541	0.292	1.125	0.264	9.00	0.621
Our primary care team uses electronic data (e.g. diagnostical findings, lab results), to track quality indicators of patient care, e.g. percentage of patients with type 2 diabetes mellitus whose HbA1C was checked last year.		121	1.77	1.101	1.213	1.501	1.498	19.25	0.544
Our primary care team uses an electronic patient chart or other electronic system, to support the documentation of patient needs.		136	1.24	0.683	0.467	3.614	14.701	6.00	0.305
Our primary care team uses an electronic health record system or other electronic systems to develop care plans.		125	2.55	1.405	1.975	0.485	−1.094	38.75	0.529
Our primary care team uses an electronic health record system or other electronic systems to determine clinical outcomes.		136	1.63	0.949	0.901	1.749	2.842	15.75	0.462
Our primary care team informs patients about any diagnosis in a way that they can understand.	Communication	136	1.60	0.837	0.700	1.708	3.477	15.00	0.475
Someone on our primary care team helps patients understand all of the choices for their care.		135	1.44	0.665	0.443	1.518	2.244	11.00	0.695
Our primary care team considers and respects patients’ individual values when recommending treatments.		135	1.65	0.716	0.512	1.000	0.991	16.25	0.553
Our primary care team’s care coordination activities are based upon ongoing assessment of patient needs.		128	1.75	0.896	0.803	1.317	1.813	18.75	0.408
Someone on our primary care team asks for patients‘ input when making a plan for their care.	Plan of Care	39	1.97	0.932	0.868	0.876	0.165	24.25	0.704
Someone on our primary care team helps make care plans that patients can follow in their daily life.		41	1.93	0.818	0.670	1.002	1.184	23.25	0.811
Someone on our primary care team develops care plans that incorporate plans recommended by other health care providers that patients see.		40	1.90	0.709	0.503	0.600	0.779	22.50	0.487
Our primary care team reviews and updates patients‘ care plans with them.	Follow-up Plan of Care	42	1.57	0.737	0.544	1.276	1.569	14.25	0.524
Our primary care team gives patients a copy of their care plan.^M^		40	1.55	0.677	0.459	0.852	−0.368	13.75	0.308
Our primary care team follows through with the care plans.		42	1.71	0.774	0.599	0.885	0.385	17.75	0.598
Our primary care team uses patients‘ care plans to follow progress.		39	1.82	0.644	0.414	0.177	−0.534	20.50	0.596
Our primary care team helps patients plan so they can take care of their health even when things change or when unexpected things happen.		133	1.68	0.792	0.627	1.001	0.457	17.00	0.288
Someone on our primary care team helps patients set goals for managing their health.	Self-Management	132	1.93	0.764	0.583	0.429	−0.306	23.25	0.489
Someone on our primary care team checks to see if patients are reaching their goals.		128	2.00	0.851	0.724	0.701	0.451	25.00	0.618
Our practice has behaviour change interventions readily available for patients as part of routine care.		132	2.01	1.149	1.321	1.119	0.387	25.25	0.548
Our practice has peer support readily available for patients as part of routine care.		119	3.71	1.311	1.718	−0.586	−0.833	67.75	0.492
Someone on our primary care team asks patients about what they need for support, e.g. with home care, equipment or transportation.	Link to Community Resources	137	1.74	0.885	0.783	1.189	0.781	18.50	0.582
Someone on our primary care team offers patients the opportunity to learn more about managing their health, e.g. with group appointments, support groups, or patient education.		134	2.16	1.343	1.802	0.977	−0.334	29.00	0.412
Someone on our primary care team gives patients information about additional supportive services offered at the practice or in their community, e.g. counselling programmes, support groups or rehabilitation programmes.		136	1.76	0.960	0.922	1.457	1.959	19.00	0.650
Someone on our primary care team encourages patients to attend programmes in their community, e.g. support or sports groups.		134	1.65	0.895	0.801	1.585	2.514	16.25	0.720
Someone on our primary care team connects patients to needed services, e.g. transportation or home care.		135	1.36	0.592	0.350	1.676	2.849	9.00	0.554
When patients are discharged from the hospital, our primary care team is informed about the care patients received from the hospital.	Care Transitions	134	1.71	0.812	0.659	1.267	1.946	17.75	0.604
When patients are discharged from the hospital, our primary care team knows about new prescriptions or if there was a change in medication.		136	1.57	0.737	0.543	1.660	4.088	14.25	0.727
When patients are discharged from hospital, our primary care team receives the discharge summaries in a timely manner.		136	1.90	0.702	0.493	0.797	1.356	22.50	0.592
When patients are discharged from the hospital and there are test results pending, they will be integrated into the patient records within two weeks.		137	2.15	0.817	0.758	0.796	0.513	28.75	0.425

* observed in original domain

^N^ missing in analysis results description in original publication, included in final questionnaire of original publication, domain IT Capacity assumed

^M^ missing in analysis results description in original publication, included in final questionnaire of original publication, domain Follow-up Plan of Care assumed

An exploratory factor analysis with all items and listwise deletion of cases included 23 datasets which is insufficient to describe 35 items. The approach of the replacement of missing values with the variable mean resulted in the inclusion of 140 datasets. This factor analysis showed KMO = 0.790 and Bartlett’s test = 1.000. MSA were lowest for the items “our primary care team gives patients a copy of their care plan” (MSA = 0.431), “our primary care team follows through with the care plans” (MSA = 0.589), “our primary care team uses patients‘ care plans to follow progress” (MSA = 0.616), “someone on our primary care team asks for patients‘ input when making a plan for their care” (MSA = 0.664), “someone on our primary care team helps make care plans that patients can follow in their daily life” (MSA = 0.688), and “when patients are discharged from the hospital and there are test results pending, they will be integrated into the patient records within two weeks” (MSA = 0.685). As a result of those values and the high number of missings in the items of the original domains “plan of care” and “follow-up plan of care” all items regarding the original domains “plan of care” and “follow-up plan of care” and the item “when patients are discharged from the hospital and there are test results pending, they will be integrated into the patient records within two weeks” were excluded from factor analysis. The exploratory factor analysis of the remaining 27 items with listwise deletion of cases with missing values included 88 observations. The Bartlett’s test of sphericity was significant (< 0.001) for all domains, the Kaiser-Meyer-Olkin measure of sampling adequacy (KMO = 0.828) indicated that the sample was suitable for factor analysis. It resulted in the seven domains “accountability”, “information technology for quality assurance”, “information technology supporting patient care”, “self-management”, “communication”, “link to community resources” and “care transitions”. Three items were assigned to other domains in comparison to the original validation study; the domain “IT capacity” was split into “information technology for quality assurance” and “information technology supporting patient care”. Cronbach’s α was acceptable for the domains “accountability” (α = 0.698), “information technology for quality assurance” (α = 0.680), “information technology supporting patient care” (α = 0.690), and “self-management” (α = 0.732), and good for the domains “care transitions” (α = 0.808), “communication” (α = 0.815), and “link to community resources” (α = 0.819). Factor loadings (λ), explained variances (R^2^) and Cronbach’s α are shown in Table 6.

**Table 6:** Structure of the final 7 domain German healthcare team survey as emerged from analysis (27 items, Cronbach’s α = 0.926)

Domain	Items	Factor analysis with listwise deletion of cases with missing values, ***N*** = 88, eigenvalue > 1, KMO = 0,828, Bartlett’s test < 0.001		
		MSA	λ	R^**2**^
**Communication (α = 0.815)**	*Someone on our primary care team helps patients set goals for managing their health.*^N^	0.822	0.732	0.727
	Someone on our primary care team helps patients understand all of the choices for their care.	0.900	0.702	0.800
	*Our primary care team helps patients plan so they can take care of their health even when things change or when unexpected things happen*.^M^	0.835	0.681	0.720
	Our primary care team considers and respects patients’ individual values when recommending treatments.	0.845	0.641	0.685
	Our primary care team informs patients about any diagnosis in a way that they can understand.	0.851	0.634	0.687
	Our primary care team’s care coordination activities are based upon ongoing assessment of patient needs.	0.893	0.522	0.517
**Link to Community Resources (α = 0.819)**	Someone on our primary care team encourages patients to attend programmes in their community, e.g. support or sports groups.	0.782	0.796	0.817
	Someone on our primary care team gives patients information about additional supportive services offered at the practice or in their community, e.g. counselling programmes, support groups or rehabilitation programmes.	0.790	0.771	0.815
	Someone on our primary care team connects patients to needed services, e.g. transportation or home care.	0.858	0.753	0.690
	Someone on our primary care team asks patients about what they need for support, e.g. with home care, equipment or transportation.	0.944	0.392	0.665
**Information Technology for Quality Assurance (α = 0.680)**	*Our primary care team uses electronic data (*e.g. *diagnostical findings, lab results), to track quality indicators of patient care,* e.g. *percentage of patients with type 2 diabetes mellitus whose HbA1C was checked last year.*^P^	0.758	0.720	0.728
	*Our primary care team uses an electronic health record system or other electronic systems to determine clinical outcomes.*^P^	0.845	0.613	0.677
	*Our primary care team uses an electronic health record system or other electronic systems to develop care plans.*^P^	0.856	0.585	0.698
**Self-Management (α = 0.732)**	Our practice has peer support readily available for patients as part of routine care.	0.849	0.714	0.642
	*Someone on our primary care team offers patients the opportunity to learn more about managing their health,* e.g. *with group appointments, support groups, or patient education.*^O^	0.816	0.657	0.694
	Someone on our primary care team checks to see if patients are reaching their goals.	0.913	0.548	0.734
	Our practice has behaviour change interventions readily available for patients as part of routine care.	0.834	0.530	0.650
**Care Transitions (α = 0.808)**	When patients are discharged from the hospital, our primary care team knows about new prescriptions or if there was a change in medication.	0.723	0.849	0.876
	When patients are discharged from the hospital, our primary care team is informed about the care patients received from the hospital.	0.770	0.833	0.826
	When patients are discharged from hospital, our primary care team receives the discharge summaries in a timely manner.	0.773	0.687	0.719
**Accountability (α = 0.698)**	Our primary care team is made up of members with clearly defined roles in caring for patients.	0.723	0.776	0.691
	Our primary care team and the patients share responsibilities in managing patients’ health.	0.661	0.728	0.669
	Our primary care team works with patients to help them understand their roles and responsibilities in their care.	0.855	0.587	0.740
	Our primary care team is characterised by trusting collaboration.	0.827	0.481	0.738
**Information Technology Supporting Patient Care (α = 0.690)**	*Our primary care team uses an electronic patient chart or other electronic system, to support the documentation of patient needs.*^P^	0.776	0.779	0.659
	*Our primary care team uses electronic data (*e.g. *diagnostical findings, lab results), to identify patients with complex health needs (*e.g. *multimorbity).*^P^	0.869	0.729	0.737
	*Our primary care team uses electronic data (*e.g. *diagnostical findings, lab results), to monitor the outcomes of patient treatment.*^P^	0.865	0.590	0.638

^N^ originally belonging to domain „self-management“

^M^ originally belonging to domain „follow-up plan of care“

^O^ originally belonging to domain „link to community resources“

^P^ originally belonging to domain „IT capacity“

The convergent validity was acceptable with r_rho_ ranging from 0.355 to 0.498 (*p* < 0.001) implying that the domains were associated with the general care given by the primary care team. The calculation of the discriminant validity resulted in r_rho_ between − 0.088 and 0.069 (*p* value between 0.313 and 0.849). Hence, there was no significant correlation between the gender of the participants and the domains.

## Discussion

The MHCCS-P and the MHCCS-H were successfully translated into German, culturally adapted, and examined for their psychometric properties for utilization in the German primary care system.

The instruments were developed to measure care coordination, a core element of the PCMH, from the view of the patients and the healthcare team. Exploratory factor analyses revealed a three-domain MHCCS-D-P and a seven-domain MHCCS-D-H with acceptable or good internal consistencies (α = 0.680 to α = 0.936). While many authors state α ≥ 0.7 as acceptable e.g. [37], it is also argued that this appears to be an artificial threshold [38]. Even low values of 0.5 “do not seriously attenuate validity coefficients” [39]. Scales that are designed to measure seemingly unidimensional concepts with a resulting high Cronbach’s α will have some degree of heterogeneity among the items since there are probably different aspects in these concepts [40]. Since α depends on the length of the scale [40], it can be assumed that a scale with a low number of items tends to show lower α values. All scales in this study with α < 0.8 consisted of only three or four items.

The approach of an exploratory factor analysis seemed appropriate for the testing of psychometric properties of the MHCCS-D because of the differences between the German and the American health care system, e.g. in the accessibility of the health care system in terms of the statutory regulated linkage to health care insurance. In Germany, 99.9% of the population had a health care insurance in 2015, almost 88% in statutory health care institutions [41]. In the United States of America, 91.2% of the population had a health care insurance in 2017; 37.7% were on government coverage [42]. In a comparison between the health care systems, German patients were described as having “no incentive to limit their demand” [43]. Consequences might be more doctors’ consultations in one year per capita of the German population than the population of other major developed countries as seen in the OECD data [44]. Clearly, this might affect the needs in and the status of care coordination measures in different countries. Moreover, the authors of the original publication of instrument development themselves experienced changes from hypothesized item assignment to item assignment in analyses. As a result, a solely confirmative approach seemed too restrictive in order to appreciate the efforts behind the development of the conceptual model and the establishment of content validity by the authors of the MHCCS.

The comprehensive development process of the MHCCS as well as the translation and cultural adaptation process of the MHCCS-D supports content validity of the MHCCS-D. Construct validity was established by exploratory factor analysis revealing relevant domains in the care for people with chronic diseases. Thus, the domains of the MHCCS-D “link to community resources”, “self-management”, “information technology for quality assurance” and “information technology supporting patient care” are also represented in the Chronic Care Model (CCM) [45]. It is known that interventions including at least one CCM element improve clinical outcomes and the quality of life of patients with chronic diseases [46]. Former research based on the CCM suggests that the structure of care in Germany can be improved especially by specific goal setting, care coordination and follow-up [47].

In total, 77% of patients (*n* = 232) and 63% of health care team members denied to have a written care plan or make written care plans for their patients. It might be concluded that a plan of care is not yet established in German primary care practices. Discrepancies in descriptive item analysis and factor analysis led to the exclusion of the items regarding the plan of care in the final MHCCS-D. However, the use of individual care plans has shown better clinical outcomes as well as better quality of care by primary care physicians [48, 49]. In October 2016, standard medication plans were established on a national level in Germany as a result of varying quality of plans used up to then. Patients have to get a medication plan (including information on registered trade names, active components, dose, dosage form and reminder on how to take the prescribed drugs) if they get three or more different drugs on a regular basis [50]. The further introduction of individual care plans - in addition to medication plans - that are followed through and updated on a regular basis would additionally enhance structure quality [51].

That the concepts “plan of care”, “primary care team” and “primary care practice” are not well-established in Germany was confirmed in this study, in the translation and piloting process as well as in the results, comments and remarks of participants. Hence, the applicability of these terms is not given. Currently, the instrument is being tested with relevant changes in another study.

The MHCCS-D showed acceptable convergent validity with self-reported general care given by the primary care team and with the EUROPEP, an internationally established instrument reflecting indicators for patients to evaluate their perceived quality of care. Additionally, the predictive validity of the MHCCS-D-P was proven for all domains with the self-reported coordination of care suggesting a reliable instrument in order to reflect the quality of care in three domains.

All in all, both instruments can be used in further studies to measure care coordination in German primary care practices with patients’ as well as healthcare team members’ self-assessments. Since the total number of items is low (9 for patients and 27 for team members), practicability is high.

### Strengths and limitations

The response rates of 43 and 34% were similar compared to other studies in primary care [52]. The convenient sample of patients led to a female/male-ratio that is similar with other studies performed in primary care [30, 31]. Nearly half of the participants reported having a chronic disease which appears to be higher than in the general population with about 44.2% reporting to have a long-standing illness or health problem in 2017 in Germany [53]. However, it is known that in German primary care practices more than 60% of the patients are older than 60 years and among these patients about 60% suffer from multimorbidity [54].

The authors of the original MHCCS described their patient sample as a “low-income, low-literacy patient population” with higher rates of chronic illness and poorer health outcomes. This was described as both strength and limitation of the study. Inclusion criteria of this study were not restricted to patients with chronic illness in order to test the applicability to the unselected patient population of primary care practices. This approach might lead to a better generalizability of results to a wider patient population without focusing on chronic diseases. However, the transferability of the results to primary care practices and patients in general is not guaranteed, especially because a selection bias of primary care practices is possible due to higher participation interest of practices that are already thinking of ways for improving their care coordination.

Since the patient questionnaire contained further items due to the willingness to evaluate the convergent validity with EUROPEP, healthcare team members might not have invited every patient to participate in this study consecutively, but chose patients they knew better because of regular visits to the practice. The healthcare teams might be interested in not deterring patients from coming back into the practice by burdening them with a time-consuming questionnaire.

Issues with response rate and selection of patients can be approached by repeating this study - after some time or in other areas if necessary - or administrating questionnaires by interviews.

The employed, non-physician health professionals were declared to be 3.8 per primary care practice owner in Germany in 2013 [55]. Our physician/non-physician-ratio of 3.4 is near that number.

Finally, the test-retest reliability was not measured. Therefore, no conclusions about the sensitivity to change can be drawn.

## Conclusions

The German versions of the Medical Home Care Coordination Survey for patients and health care team members make care coordination measurable in German primary care practices.

Shortcomings of the German health care system - all attributed to various coordination challenges - can be approached by measuring different aspects of care coordination with these instruments and consequently, they are revealing needs for improvement. As suggested by the authors of the original instrument development the adapted German versions are currently tested in a longitudinal approach to determine whether these instruments are capable of detecting changes in care coordination over time.

## Data Availability

The datasets used and analysed during the current study are available from the corresponding author on reasonable request.

## Abbreviations

AHRQ: Agency for Healthcare Research and Quality
MHCCS: Medical Home Care Coordination Survey
MHCCS-D: German version of the Medical Home Care Coordination Survey
MHCCS-D-H: German version of the Medical Home Care Coordination Survey - assessment by healthcare team
MHCCS-D-P: German version of the Medical Home Care Coordination Survey - assessment by patient
MHCCS-H: Medical Home Care Coordination Survey – assessment by healthcare team
MHCCS-P: Medical Home Care Coordination Survey – assessment by patient
ISPOR: International Society for Pharmacoeconomics and Outcomes Research
EUROPEP: European Project on Patient Evaluation of General Practice Care
